# Supplementary material for: Resolving the full spectrum of human genome variation using Linked-Reads
Source: Genome Res. 2019 Apr;29(4):635–45. doi: 10.1101/gr.234443.118 (PMC6442396; doi:10.1101/gr.234443.118)
Supplement: Supplemental Material [file supp_gr.234443.118_Supplemental_longranger-2.2.zip › longranger-2.2.tar.bz2!/lr2.2-public/lib/stan/cmdstan/stan/doc/api/html/var__decls__grammar__def_8hpp_source.html]

Stan: src/stan/gm/grammars/var\_decls\_grammar\_def.hpp Source File


|  |  |
| --- | --- |
|  | Stan  2.5.0  probability, sampling & optimization |


- Main Page
- Related Pages
- Namespaces
- Classes
- Files

- File List
- File Members

All Classes Namespaces Files Functions Variables Typedefs Enumerations Enumerator Friends Macros Pages

- src
- stan
- gm
- grammars

var\_decls\_grammar\_def.hpp

Go to the documentation of this file.

1 #ifndef STAN\_\_GM\_\_PARSER\_\_VAR\_DECLS\_GRAMMAR\_DEF\_\_HPP

2 #define STAN\_\_GM\_\_PARSER\_\_VAR\_DECLS\_GRAMMAR\_DEF\_\_HPP

3

4 #include <boost/spirit/include/qi.hpp>

5 // FIXME: get rid of unused include

6 #include <boost/spirit/include/phoenix\_core.hpp>

7 #include <boost/spirit/include/phoenix\_function.hpp>

8 #include <boost/spirit/include/phoenix\_fusion.hpp>

9 #include <boost/spirit/include/phoenix\_object.hpp>

10 #include <boost/spirit/include/phoenix\_operator.hpp>

11 #include <boost/spirit/include/phoenix\_stl.hpp>

12

13 #include <boost/lexical\_cast.hpp>

14 #include <boost/fusion/include/adapt\_struct.hpp>

15 #include <boost/fusion/include/std\_pair.hpp>

16 #include <boost/config/warning\_disable.hpp>

17 #include <boost/spirit/include/qi\_numeric.hpp>

18 #include <stan/gm/ast.hpp>

19 #include <stan/gm/grammars/var\_decls\_grammar.hpp>

20 #include <stan/gm/grammars/common\_adaptors\_def.hpp>

21

22 BOOST\_FUSION\_ADAPT\_STRUCT(stan::gm::int\_var\_decl,

23  (stan::gm::range, range\_)

24  (std::string, name\_)

25  (std::vector<stan::gm::expression>, dims\_) )

26

27 BOOST\_FUSION\_ADAPT\_STRUCT(stan::gm::double\_var\_decl,

28  (stan::gm::range, range\_)

29  (std::string, name\_)

30  (std::vector<stan::gm::expression>, dims\_) )

31

32 BOOST\_FUSION\_ADAPT\_STRUCT(stan::gm::vector\_var\_decl,

33  (stan::gm::range, range\_)

34  (stan::gm::expression, M\_)

35  (std::string, name\_)

36  (std::vector<stan::gm::expression>, dims\_) )

37

38 BOOST\_FUSION\_ADAPT\_STRUCT(stan::gm::row\_vector\_var\_decl,

39  (stan::gm::range, range\_)

40  (stan::gm::expression, N\_)

41  (std::string, name\_)

42  (std::vector<stan::gm::expression>, dims\_) )

43

44 BOOST\_FUSION\_ADAPT\_STRUCT(stan::gm::matrix\_var\_decl,

45  (stan::gm::range, range\_)

46  (stan::gm::expression, M\_)

47  (stan::gm::expression, N\_)

48  (std::string, name\_)

49  (std::vector<stan::gm::expression>, dims\_) )

50

51 BOOST\_FUSION\_ADAPT\_STRUCT(stan::gm::unit\_vector\_var\_decl,

52  (stan::gm::expression, K\_)

53  (std::string, name\_)

54  (std::vector<stan::gm::expression>, dims\_) )

55

56 BOOST\_FUSION\_ADAPT\_STRUCT(stan::gm::simplex\_var\_decl,

57  (stan::gm::expression, K\_)

58  (std::string, name\_)

59  (std::vector<stan::gm::expression>, dims\_) )

60

61 BOOST\_FUSION\_ADAPT\_STRUCT(stan::gm::ordered\_var\_decl,

62  (stan::gm::expression, K\_)

63  (std::string, name\_)

64  (std::vector<stan::gm::expression>, dims\_) )

65

66 BOOST\_FUSION\_ADAPT\_STRUCT(stan::gm::positive\_ordered\_var\_decl,

67  (stan::gm::expression, K\_)

68  (std::string, name\_)

69  (std::vector<stan::gm::expression>, dims\_) )

70

71 BOOST\_FUSION\_ADAPT\_STRUCT(stan::gm::cholesky\_factor\_var\_decl,

72  (stan::gm::expression, M\_)

73  (stan::gm::expression, N\_)

74  (std::string, name\_)

75  (std::vector<stan::gm::expression>, dims\_) )

76

77 BOOST\_FUSION\_ADAPT\_STRUCT(stan::gm::cholesky\_corr\_var\_decl,

78  (stan::gm::expression, K\_)

79  (std::string, name\_)

80  (std::vector<stan::gm::expression>, dims\_) )

81

82 BOOST\_FUSION\_ADAPT\_STRUCT(stan::gm::cov\_matrix\_var\_decl,

83  (stan::gm::expression, K\_)

84  (std::string, name\_)

85  (std::vector<stan::gm::expression>, dims\_) )

86

87 BOOST\_FUSION\_ADAPT\_STRUCT(stan::gm::corr\_matrix\_var\_decl,

88  (stan::gm::expression, K\_)

89  (std::string, name\_)

90  (std::vector<stan::gm::expression>, dims\_) )

91

92 namespace stan {

93

94  namespace gm {

95

96  struct validate\_no\_constraints\_vis : public boost::static\_visitor<bool> {

97  std::stringstream& error\_msgs\_;

98  validate\_no\_constraints\_vis(std::stringstream& error\_msgs)

99  : error\_msgs\_(error\_msgs) {

100  }

101  bool operator()(const nil& /\*x\*/) const {

102  error\_msgs\_ << "nil declarations not allowed";

103  return false; // fail if arises

104  }

105  bool operator()(const int\_var\_decl& x) const {

106  if (x.range\_.has\_low() || x.range\_.has\_high()) {

107  error\_msgs\_ << "require unconstrained."

108  << " found range constraint." << std::endl;

109  return false;

110  }

111  return true;

112  }

113  bool operator()(const double\_var\_decl& x) const {

114  if (x.range\_.has\_low() || x.range\_.has\_high()) {

115  error\_msgs\_ << "require unconstrained."

116  << " found range constraint." << std::endl;

117  return false;

118  }

119  return true;

120  }

121  bool operator()(const vector\_var\_decl& /\*x\*/) const {

122  return true;

123  }

124  bool operator()(const row\_vector\_var\_decl& /\*x\*/) const {

125  return true;

126  }

127  bool operator()(const matrix\_var\_decl& /\*x\*/) const {

128  return true;

129  }

130  bool operator()(const unit\_vector\_var\_decl& /\*x\*/) const {

131  error\_msgs\_ << "require unconstrained variable declaration."

132  << " found unit\_vector." << std::endl;

133  return false;

134  }

135  bool operator()(const simplex\_var\_decl& /\*x\*/) const {

136  error\_msgs\_ << "require unconstrained variable declaration."

137  << " found simplex." << std::endl;

138  return false;

139  }

140  bool operator()(const ordered\_var\_decl& /\*x\*/) const {

141  error\_msgs\_ << "require unconstrained variable declaration."

142  << " found ordered." << std::endl;

143  return false;

144  }

145  bool operator()(const positive\_ordered\_var\_decl& /\*x\*/) const {

146  error\_msgs\_ << "require unconstrained variable declaration."

147  << " found positive\_ordered." << std::endl;

148  return false;

149  }

150  bool operator()(const cholesky\_factor\_var\_decl& /\*x\*/) const {

151  error\_msgs\_ << "require unconstrained variable declaration."

152  << " found cholesky\_factor." << std::endl;

153  return false;

154  }

155  bool operator()(const cholesky\_corr\_var\_decl& /\*x\*/) const {

156  error\_msgs\_ << "require unconstrained variable declaration."

157  << " found cholesky\_factor\_corr." << std::endl;

158  return false;

159  }

160  bool operator()(const cov\_matrix\_var\_decl& /\*x\*/) const {

161  error\_msgs\_ << "require unconstrained variable declaration."

162  << " found cov\_matrix." << std::endl;

163  return false;

164  }

165  bool operator()(const corr\_matrix\_var\_decl& /\*x\*/) const {

166  error\_msgs\_ << "require unconstrained variable declaration."

167  << " found corr\_matrix." << std::endl;

168  return false;

169  }

170  };

171

172  struct data\_only\_expression : public boost::static\_visitor<bool> {

173  std::stringstream& error\_msgs\_;

174  variable\_map& var\_map\_;

175  data\_only\_expression(std::stringstream& error\_msgs,

176  variable\_map& var\_map)

177  : error\_msgs\_(error\_msgs),

178  var\_map\_(var\_map) {

179  }

180  bool operator()(const nil& /\*e\*/) const {

181  return true;

182  }

183  bool operator()(const int\_literal& /\*x\*/) const {

184  return true;

185  }

186  bool operator()(const double\_literal& /\*x\*/) const {

187  return true;

188  }

189  bool operator()(const array\_literal& x) const {

190  for (size\_t i = 0; i < x.args\_.size(); ++i)

191  if (!boost::apply\_visitor(\*this,x.args\_[i].expr\_))

192  return false;

193  return true;

194  }

195  bool operator()(const variable& x) const {

196  var\_origin origin = var\_map\_.get\_origin(x.name\_);

197  bool is\_data = (origin == data\_origin)

198  || (origin == transformed\_data\_origin)

199  || (origin == local\_origin);

200  if (!is\_data) {

201  error\_msgs\_ << "non-data variables not allowed in dimension declarations."

202  << std::endl

203  << " found variable=" << x.name\_

204  << "; declared in block=";

205  print\_var\_origin(error\_msgs\_,origin);

206  error\_msgs\_ << std::endl;

207  }

208  return is\_data;

209  }

210  bool operator()(const integrate\_ode& x) const {

211  return boost::apply\_visitor(\*this, x.y0\_.expr\_)

212  && boost::apply\_visitor(\*this, x.theta\_.expr\_);

213  }

214  bool operator()(const fun& x) const {

215  for (size\_t i = 0; i < x.args\_.size(); ++i)

216  if (!boost::apply\_visitor(\*this,x.args\_[i].expr\_))

217  return false;

218  return true;

219  }

220  bool operator()(const index\_op& x) const {

221  if (!boost::apply\_visitor(\*this,x.expr\_.expr\_))

222  return false;

223  for (size\_t i = 0; i < x.dimss\_.size(); ++i)

224  for (size\_t j = 0; j < x.dimss\_[i].size(); ++j)

225  if (!boost::apply\_visitor(\*this,x.dimss\_[i][j].expr\_))

226  return false;

227  return true;

228  }

229  bool operator()(const binary\_op& x) const {

230  return boost::apply\_visitor(\*this,x.left.expr\_)

231  && boost::apply\_visitor(\*this,x.right.expr\_);

232  }

233  bool operator()(const unary\_op& x) const {

234  return boost::apply\_visitor(\*this,x.subject.expr\_);

235  }

236  };

237

238

239  struct add\_var {

240  template <typename T1, typename T2, typename T3, typename T4, typename T5, typename T6>

241  struct result { typedef void type; };

242  // each type derived from base\_var\_decl gets own instance

243  template <typename R, typename T>

244  void operator()(R& var\_decl\_result,

245  const T& var\_decl,

246  variable\_map& vm,

247  bool& pass,

248  const var\_origin& vo,

249  std::ostream& error\_msgs) const {

250  if (vm.exists(var\_decl.name\_)) {

251  // variable already exists

252  pass = false;

253  error\_msgs << "duplicate declaration of variable, name="

254  << var\_decl.name\_;

255

256  error\_msgs << "; attempt to redeclare as ";

257  print\_var\_origin(error\_msgs,vo); // FIXME -- need original vo

258

259  error\_msgs << "; original declaration as ";

260  print\_var\_origin(error\_msgs,vm.get\_origin(var\_decl.name\_));

261

262  error\_msgs << std::endl;

263  var\_decl\_result = var\_decl;

264  return;

265  }

266  if ((vo == parameter\_origin || vo == transformed\_parameter\_origin)

267  && var\_decl.base\_type\_ == INT\_T) {

268  pass = false;

269  error\_msgs << "integer parameters or transformed parameters are not allowed; "

270  << " found declared type int, parameter name=" << var\_decl.name\_

271  << std::endl;

272  var\_decl\_result = var\_decl;

273  return;

274  }

275  pass = true; // probably don't need to set true

276  vm.add(var\_decl.name\_,var\_decl,vo);

277  var\_decl\_result = var\_decl;

278  }

279  };

280  boost::phoenix::function<add\_var> add\_var\_f;

281

282

283  struct validate\_decl\_constraints {

284  template <typename T1, typename T2, typename T3, typename T4>

285  struct result { typedef bool type; };

286

287  bool operator()(const bool& allow\_constraints,

288  const bool& declaration\_ok,

289  const var\_decl& var\_decl,

290  std::stringstream& error\_msgs) const {

291  if (!declaration\_ok) {

292  error\_msgs << "Problem with declaration." << std::endl;

293  return false; // short-circuits test of constraints

294  }

295  if (allow\_constraints)

296  return true;

297  validate\_no\_constraints\_vis vis(error\_msgs);

298  bool constraints\_ok = boost::apply\_visitor(vis,var\_decl.decl\_);

299  return constraints\_ok;

300  }

301  };

302  boost::phoenix::function<validate\_decl\_constraints>

303  validate\_decl\_constraints\_f;

304

305  struct validate\_identifier {

306  std::set<std::string> reserved\_word\_set\_;

307  std::set<std::string> const\_fun\_name\_set\_;

308

309  template <typename T1, typename T2>

310  struct result { typedef bool type; };

311

312  void reserve(const std::string& w) {

313  reserved\_word\_set\_.insert(w);

314  }

315

316  template <typename S, typename T>

317  static bool contains(const S& s,

318  const T& x) {

319  return s.find(x) != s.end();

320  }

321

322  bool identifier\_exists(const std::string& identifier) const {

323  return contains(reserved\_word\_set\_, identifier)

324  || ( contains(function\_signatures::instance().key\_set(), identifier)

325  && !contains(const\_fun\_name\_set\_, identifier) );

326  }

327

328  validate\_identifier() {

329  // Constant functions which can be used as identifiers

330  const\_fun\_name\_set\_.insert("pi");

331  const\_fun\_name\_set\_.insert("e");

332  const\_fun\_name\_set\_.insert("sqrt2");

333  const\_fun\_name\_set\_.insert("log2");

334  const\_fun\_name\_set\_.insert("log10");

335  const\_fun\_name\_set\_.insert("not\_a\_number");

336  const\_fun\_name\_set\_.insert("positive\_infinity");

337  const\_fun\_name\_set\_.insert("negative\_infinity");

338  const\_fun\_name\_set\_.insert("epsilon");

339  const\_fun\_name\_set\_.insert("negative\_epsilon");

340

341  // illegal identifiers

342  reserve("for");

343  reserve("in");

344  reserve("while");

345  reserve("repeat");

346  reserve("until");

347  reserve("if");

348  reserve("then");

349  reserve("else");

350  reserve("true");

351  reserve("false");

352

353  reserve("int");

354  reserve("real");

355  reserve("vector");

356  reserve("unit\_vector");

357  reserve("simplex");

358  reserve("ordered");

359  reserve("positive\_ordered");

360  reserve("row\_vector");

361  reserve("matrix");

362  reserve("cholesky\_factor\_cov");

363  reserve("cholesky\_factor\_corr");

364  reserve("cov\_matrix");

365  reserve("corr\_matrix");

366

367

368  reserve("model");

369  reserve("data");

370  reserve("parameters");

371  reserve("quantities");

372  reserve("transformed");

373  reserve("generated");

374

375  reserve("var");

376

377  reserve("alignas");

378  reserve("alignof");

379  reserve("and");

380  reserve("and\_eq");

381  reserve("asm");

382  reserve("auto");

383  reserve("bitand");

384  reserve("bitor");

385  reserve("bool");

386  reserve("break");

387  reserve("case");

388  reserve("catch");

389  reserve("char");

390  reserve("char16\_t");

391  reserve("char32\_t");

392  reserve("class");

393  reserve("compl");

394  reserve("const");

395  reserve("constexpr");

396  reserve("const\_cast");

397  reserve("continue");

398  reserve("decltype");

399  reserve("default");

400  reserve("delete");

401  reserve("do");

402  reserve("double");

403  reserve("dynamic\_cast");

404  reserve("else");

405  reserve("enum");

406  reserve("explicit");

407  reserve("export");

408  reserve("extern");

409  reserve("false");

410  reserve("float");

411  reserve("for");

412  reserve("friend");

413  reserve("goto");

414  reserve("if");

415  reserve("inline");

416  reserve("int");

417  reserve("long");

418  reserve("mutable");

419  reserve("namespace");

420  reserve("new");

421  reserve("noexcept");

422  reserve("not");

423  reserve("not\_eq");

424  reserve("nullptr");

425  reserve("operator");

426  reserve("or");

427  reserve("or\_eq");

428  reserve("private");

429  reserve("protected");

430  reserve("public");

431  reserve("register");

432  reserve("reinterpret\_cast");

433  reserve("return");

434  reserve("short");

435  reserve("signed");

436  reserve("sizeof");

437  reserve("static");

438  reserve("static\_assert");

439  reserve("static\_cast");

440  reserve("struct");

441  reserve("switch");

442  reserve("template");

443  reserve("this");

444  reserve("thread\_local");

445  reserve("throw");

446  reserve("true");

447  reserve("try");

448  reserve("typedef");

449  reserve("typeid");

450  reserve("typename");

451  reserve("union");

452  reserve("unsigned");

453  reserve("using");

454  reserve("virtual");

455  reserve("void");

456  reserve("volatile");

457  reserve("wchar\_t");

458  reserve("while");

459  reserve("xor");

460  reserve("xor\_eq");

461

462  // function names declared in signatures

463  using stan::gm::function\_signatures;

464  using std::set;

465  using std::string;

466  const function\_signatures& sigs = function\_signatures::instance();

467

468  set<string> fun\_names = sigs.key\_set();

469  for (set<string>::iterator it = fun\_names.begin(); it != fun\_names.end(); ++it)

470  if (!contains(const\_fun\_name\_set\_, \*it))

471  reserve(\*it);

472  }

473

474  bool operator()(const std::string& identifier,

475  std::stringstream& error\_msgs) const {

476  int len = identifier.size();

477  if (len >= 2

478  && identifier[len-1] == '\_'

479  && identifier[len-2] == '\_') {

480  error\_msgs << "variable identifier (name) may not end in double underscore (\_\_)"

481  << std::endl

482  << " found identifer=" << identifier << std::endl;

483  return false;

484  }

485  size\_t period\_position = identifier.find('.');

486  if (period\_position != std::string::npos) {

487  error\_msgs << "variable identifier may not contain a period (.)"

488  << std::endl

489  << " found period at position (indexed from 0)=" << period\_position

490  << std::endl

491  << " found identifier=" << identifier

492  << std::endl;

493  return false;

494  }

495  if (identifier\_exists(identifier)) {

496  error\_msgs << "variable identifier (name) may not be reserved word"

497  << std::endl

498  << " found identifier=" << identifier

499  << std::endl;

500  return false;

501  }

502  return true;

503  }

504  };

505  boost::phoenix::function<validate\_identifier> validate\_identifier\_f;

506

507  // copies single dimension from M to N if only M declared

508  struct copy\_square\_cholesky\_dimension\_if\_necessary {

509  template <typename T1>

510  struct result { typedef void type; };

511  void operator()(cholesky\_factor\_var\_decl& var\_decl) const {

512  if (is\_nil(var\_decl.N\_))

513  var\_decl.N\_ = var\_decl.M\_;

514  }

515  };

516  boost::phoenix::function<copy\_square\_cholesky\_dimension\_if\_necessary>

517  copy\_square\_cholesky\_dimension\_if\_necessary\_f;

518

519  struct empty\_range {

520  template <typename T1>

521  struct result { typedef range type; };

522  range operator()(std::stringstream& /\*error\_msgs\*/) const {

523  return range();

524  }

525  };

526  boost::phoenix::function<empty\_range> empty\_range\_f;

527

528  struct validate\_int\_expr {

529  template <typename T1, typename T2, typename T3>

530  struct result { typedef void type; };

531

532  void operator()(const expression& expr,

533  bool& pass,

534  std::stringstream& error\_msgs) const {

535  if (!expr.expression\_type().is\_primitive\_int()) {

536  error\_msgs << "expression denoting integer required; found type="

537  << expr.expression\_type() << std::endl;

538

539  pass = false;

540  return;

541  }

542  pass = true;

543  }

544  };

545  boost::phoenix::function<validate\_int\_expr> validate\_int\_expr\_f;

546

547  struct set\_int\_range\_lower {

548  template <typename T1, typename T2, typename T3, typename T4>

549  struct result { typedef void type; };

550  void operator()(range& range,

551  const expression& expr,

552  bool& pass,

553  std::stringstream& error\_msgs) const {

554  range.low\_ = expr;

555  validate\_int\_expr validator;

556  validator(expr,pass,error\_msgs);

557  }

558  };

559  boost::phoenix::function<set\_int\_range\_lower> set\_int\_range\_lower\_f;

560

561  struct set\_int\_range\_upper {

562  template <typename T1, typename T2, typename T3, typename T4>

563  struct result { typedef void type; };

564  void operator()(range& range,

565  const expression& expr,

566  bool& pass,

567  std::stringstream& error\_msgs) const {

568  range.high\_ = expr;

569  validate\_int\_expr validator;

570  validator(expr,pass,error\_msgs);

571  }

572  };

573  boost::phoenix::function<set\_int\_range\_upper> set\_int\_range\_upper\_f;

574

575

576

577  struct validate\_int\_data\_expr {

578  template <typename T1, typename T2, typename T3, typename T4, typename T5>

579  struct result { typedef void type; };

580

581  void operator()(const expression& expr,

582  int var\_origin,

583  bool& pass,

584  variable\_map& var\_map,

585  std::stringstream& error\_msgs) const {

586  if (!expr.expression\_type().is\_primitive\_int()) {

587  error\_msgs << "dimension declaration requires expression denoting integer;"

588  << " found type="

589  << expr.expression\_type()

590  << std::endl;

591  pass = false;

592  } else if (var\_origin != local\_origin) {

593  data\_only\_expression vis(error\_msgs,var\_map);

594  bool only\_data\_dimensions = boost::apply\_visitor(vis,expr.expr\_);

595  pass = only\_data\_dimensions;

596  } else {

597  // don't need to check data vs. parameter in dimensions for

598  // local variable declarations

599  pass = true;

600  }

601  }

602  };

603  boost::phoenix::function<validate\_int\_data\_expr> validate\_int\_data\_expr\_f;

604

605  struct validate\_double\_expr {

606  template <typename T1, typename T2>

607  struct result { typedef bool type; };

608

609  bool operator()(const expression& expr,

610  std::stringstream& error\_msgs) const {

611  if (!expr.expression\_type().is\_primitive\_double()

612  && !expr.expression\_type().is\_primitive\_int()) {

613  error\_msgs << "expression denoting real required; found type="

614  << expr.expression\_type() << std::endl;

615  return false;

616  }

617  return true;

618  }

619  };

620  boost::phoenix::function<validate\_double\_expr> validate\_double\_expr\_f;

621

622

623  struct set\_double\_range\_lower {

624  template <typename T1, typename T2, typename T3, typename T4>

625  struct result { typedef void type; };

626  void operator()(range& range,

627  const expression& expr,

628  bool& pass,

629  std::stringstream& error\_msgs) const {

630  range.low\_ = expr;

631  validate\_double\_expr validator;

632  pass = validator(expr,error\_msgs);

633  }

634  };

635  boost::phoenix::function<set\_double\_range\_lower> set\_double\_range\_lower\_f;

636

637  struct set\_double\_range\_upper {

638  template <typename T1, typename T2, typename T3, typename T4>

639  struct result { typedef void type; };

640  void operator()(range& range,

641  const expression& expr,

642  bool& pass,

643  std::stringstream& error\_msgs) const {

644  range.high\_ = expr;

645  validate\_double\_expr validator;

646  pass = validator(expr,error\_msgs);

647  }

648  };

649  boost::phoenix::function<set\_double\_range\_upper> set\_double\_range\_upper\_f;

650

651

652  template <typename Iterator>

653  var\_decls\_grammar<Iterator>::var\_decls\_grammar(variable\_map& var\_map,

654  std::stringstream& error\_msgs)

655  : var\_decls\_grammar::base\_type(var\_decls\_r),

656  var\_map\_(var\_map),

657  error\_msgs\_(error\_msgs),

658  // expression\_g allows full recursion

659  expression\_g(var\_map,error\_msgs),

660  // expression07\_g disallows comparisons

661  expression07\_g(var\_map,error\_msgs,expression\_g)

662  {

663

664  using boost::spirit::qi::\_1;

665  using boost::spirit::qi::\_3;

666  using boost::spirit::qi::char\_;

667  using boost::spirit::qi::eps;

668  using boost::spirit::qi::lexeme;

669  using boost::spirit::qi::lit;

670  using boost::spirit::qi::no\_skip;

671  using boost::spirit::qi::\_pass;

672  using boost::spirit::qi::\_val;

673  using boost::spirit::qi::labels::\_a;

674  using boost::spirit::qi::labels::\_r1;

675  using boost::spirit::qi::labels::\_r2;

676

677  var\_decls\_r.name("variable declarations");

678  var\_decls\_r

679  %= \*(var\_decl\_r(\_r1,\_r2))

680  ;

681

682  // \_a = error state local,

683  // \_r1 constraints allowed inherited,

684  // \_r2 var\_origin

685  var\_decl\_r.name("variable declaration");

686  var\_decl\_r

687  %= (int\_decl\_r(\_r2)

688  [add\_var\_f(\_val,\_1,boost::phoenix::ref(var\_map\_),\_a,\_r2,

689  boost::phoenix::ref(error\_msgs))]

690  | double\_decl\_r(\_r2)

691  [add\_var\_f(\_val,\_1,boost::phoenix::ref(var\_map\_),\_a,\_r2,

692  boost::phoenix::ref(error\_msgs\_))]

693  | vector\_decl\_r(\_r2)

694  [add\_var\_f(\_val,\_1,boost::phoenix::ref(var\_map\_),\_a,\_r2,

695  boost::phoenix::ref(error\_msgs\_))]

696  | row\_vector\_decl\_r(\_r2)

697  [add\_var\_f(\_val,\_1,boost::phoenix::ref(var\_map\_),\_a,\_r2,

698  boost::phoenix::ref(error\_msgs\_))]

699  | matrix\_decl\_r(\_r2)

700  [add\_var\_f(\_val,\_1,boost::phoenix::ref(var\_map\_),\_a,\_r2,

701  boost::phoenix::ref(error\_msgs\_))]

702  | unit\_vector\_decl\_r(\_r2)

703  [add\_var\_f(\_val,\_1,boost::phoenix::ref(var\_map\_),\_a,\_r2,

704  boost::phoenix::ref(error\_msgs\_))]

705  | simplex\_decl\_r(\_r2)

706  [add\_var\_f(\_val,\_1,boost::phoenix::ref(var\_map\_),\_a,\_r2,

707  boost::phoenix::ref(error\_msgs\_))]

708  | ordered\_decl\_r(\_r2)

709  [add\_var\_f(\_val,\_1,boost::phoenix::ref(var\_map\_),\_a,\_r2,

710  boost::phoenix::ref(error\_msgs\_))]

711  | positive\_ordered\_decl\_r(\_r2)

712  [add\_var\_f(\_val,\_1,boost::phoenix::ref(var\_map\_),\_a,\_r2,

713  boost::phoenix::ref(error\_msgs\_))]

714  | cholesky\_factor\_decl\_r(\_r2)

715  [add\_var\_f(\_val,\_1,boost::phoenix::ref(var\_map\_),\_a,\_r2,

716  boost::phoenix::ref(error\_msgs\_))]

717  | cholesky\_corr\_decl\_r(\_r2)

718  [add\_var\_f(\_val,\_1,boost::phoenix::ref(var\_map\_),\_a,\_r2,

719  boost::phoenix::ref(error\_msgs\_))]

720  | cov\_matrix\_decl\_r(\_r2)

721  [add\_var\_f(\_val,\_1,boost::phoenix::ref(var\_map\_),\_a,\_r2,

722  boost::phoenix::ref(error\_msgs\_))]

723  | corr\_matrix\_decl\_r(\_r2)

724  [add\_var\_f(\_val,\_1,boost::phoenix::ref(var\_map\_),\_a,\_r2,

725  boost::phoenix::ref(error\_msgs\_))]

726  )

727  > lit(';')

728  [\_pass

729  = validate\_decl\_constraints\_f(\_r1,\_a,\_val,

730  boost::phoenix::ref(error\_msgs\_))]

731  ;

732

733  int\_decl\_r.name("integer declaration");

734  int\_decl\_r

735  %= ( lit("int")

736  >> no\_skip[!char\_("a-zA-Z0-9\_")] )

737  > -range\_brackets\_int\_r(\_r1)

738  > identifier\_r

739  > opt\_dims\_r(\_r1)

740  ;

741

742  double\_decl\_r.name("real declaration");

743  double\_decl\_r

744  %= ( lit("real")

745  >> no\_skip[!char\_("a-zA-Z0-9\_")] )

746  > -range\_brackets\_double\_r(\_r1)

747  > identifier\_r

748  > opt\_dims\_r(\_r1)

749  ;

750

751  vector\_decl\_r.name("vector declaration");

752  vector\_decl\_r

753  %= ( lit("vector")

754  >> no\_skip[!char\_("a-zA-Z0-9\_")] )

755  > -range\_brackets\_double\_r(\_r1)

756  > lit('[')

757  > expression\_g(\_r1)

758  [validate\_int\_expr\_f(\_1,\_pass,boost::phoenix::ref(error\_msgs\_))]

759  > lit(']')

760  > identifier\_r

761  > opt\_dims\_r(\_r1)

762  ;

763

764  row\_vector\_decl\_r.name("row vector declaration");

765  row\_vector\_decl\_r

766  %= ( lit("row\_vector")

767  >> no\_skip[!char\_("a-zA-Z0-9\_")] )

768  > -range\_brackets\_double\_r(\_r1)

769  > lit('[')

770  > expression\_g(\_r1)

771  [validate\_int\_expr\_f(\_1,\_pass,boost::phoenix::ref(error\_msgs\_))]

772  > lit(']')

773  > identifier\_r

774  > opt\_dims\_r(\_r1)

775  ;

776

777  matrix\_decl\_r.name("matrix declaration");

778  matrix\_decl\_r

779  %= ( lit("matrix")

780  >> no\_skip[!char\_("a-zA-Z0-9\_")] )

781  > -range\_brackets\_double\_r(\_r1)

782  > lit('[')

783  > expression\_g(\_r1)

784  [validate\_int\_expr\_f(\_1,\_pass,boost::phoenix::ref(error\_msgs\_))]

785  > lit(',')

786  > expression\_g(\_r1)

787  [validate\_int\_expr\_f(\_1,\_pass,boost::phoenix::ref(error\_msgs\_))]

788  > lit(']')

789  > identifier\_r

790  > opt\_dims\_r(\_r1)

791  ;

792

793  unit\_vector\_decl\_r.name("unit\_vector declaration");

794  unit\_vector\_decl\_r

795  %= ( lit("unit\_vector")

796  >> no\_skip[!char\_("a-zA-Z0-9\_")] )

797  > lit('[')

798  > expression\_g(\_r1)

799  [validate\_int\_expr\_f(\_1,\_pass,boost::phoenix::ref(error\_msgs\_))]

800  > lit(']')

801  > identifier\_r

802  > opt\_dims\_r(\_r1)

803  ;

804

805  simplex\_decl\_r.name("simplex declaration");

806  simplex\_decl\_r

807  %= ( lit("simplex")

808  >> no\_skip[!char\_("a-zA-Z0-9\_")] )

809  > lit('[')

810  > expression\_g(\_r1)

811  [validate\_int\_expr\_f(\_1,\_pass,boost::phoenix::ref(error\_msgs\_))]

812  > lit(']')

813  > identifier\_r

814  > opt\_dims\_r(\_r1)

815  ;

816

817  ordered\_decl\_r.name("ordered declaration");

818  ordered\_decl\_r

819  %= ( lit("ordered")

820  >> no\_skip[!char\_("a-zA-Z0-9\_")] )

821  > lit('[')

822  > expression\_g(\_r1)

823  [validate\_int\_expr\_f(\_1,\_pass,boost::phoenix::ref(error\_msgs\_))]

824  > lit(']')

825  > identifier\_r

826  > opt\_dims\_r(\_r1)

827  ;

828

829  positive\_ordered\_decl\_r.name("positive\_ordered declaration");

830  positive\_ordered\_decl\_r

831  %= ( lit("positive\_ordered")

832  >> no\_skip[!char\_("a-zA-Z0-9\_")] )

833  > lit('[')

834  > expression\_g(\_r1)

835  [validate\_int\_expr\_f(\_1,\_pass,boost::phoenix::ref(error\_msgs\_))]

836  > lit(']')

837  > identifier\_r

838  > opt\_dims\_r(\_r1)

839  ;

840

841  cholesky\_factor\_decl\_r.name("cholesky factor for symmetric, positive-def declaration");

842  cholesky\_factor\_decl\_r

843  %= ( lit("cholesky\_factor\_cov")

844  >> no\_skip[!char\_("a-zA-Z0-9\_")] )

845  > lit('[')

846  > expression\_g(\_r1)

847  [validate\_int\_expr\_f(\_1,\_pass,boost::phoenix::ref(error\_msgs\_))]

848  > -( lit(',')

849  > expression\_g(\_r1)

850  [validate\_int\_expr\_f(\_1,\_pass,boost::phoenix::ref(error\_msgs\_))]

851  )

852  > lit(']')

853  > identifier\_r

854  > opt\_dims\_r(\_r1)

855  > eps

856  [copy\_square\_cholesky\_dimension\_if\_necessary\_f(\_val)]

857  ;

858

859  cholesky\_corr\_decl\_r.name("cholesky factor for correlation matrix declaration");

860  cholesky\_corr\_decl\_r

861  %= ( lit("cholesky\_factor\_corr")

862  >> no\_skip[!char\_("a-zA-Z0-9\_")] )

863  > lit('[')

864  > expression\_g(\_r1)

865  [validate\_int\_expr\_f(\_1,\_pass,boost::phoenix::ref(error\_msgs\_))]

866  > lit(']')

867  > identifier\_r

868  > opt\_dims\_r(\_r1)

869  ;

870

871  cov\_matrix\_decl\_r.name("covariance matrix declaration");

872  cov\_matrix\_decl\_r

873  %= ( lit("cov\_matrix")

874  >> no\_skip[!char\_("a-zA-Z0-9\_")] )

875  > lit('[')

876  > expression\_g(\_r1)

877  [validate\_int\_expr\_f(\_1,\_pass,boost::phoenix::ref(error\_msgs\_))]

878  > lit(']')

879  > identifier\_r

880  > opt\_dims\_r(\_r1)

881  ;

882

883  corr\_matrix\_decl\_r.name("correlation matrix declaration");

884  corr\_matrix\_decl\_r

885  %= ( lit("corr\_matrix")

886  >> no\_skip[!char\_("a-zA-Z0-9\_")] )

887  > lit('[')

888  > expression\_g(\_r1)

889  [validate\_int\_expr\_f(\_1,\_pass,boost::phoenix::ref(error\_msgs\_))]

890  > lit(']')

891  > identifier\_r

892  > opt\_dims\_r(\_r1)

893  ;

894

895  opt\_dims\_r.name("array dimensions (optional)");

896  opt\_dims\_r

897  %= - dims\_r(\_r1);

898

899  dims\_r.name("array dimensions");

900  dims\_r

901  %= lit('[')

902  > (expression\_g(\_r1)

903  [validate\_int\_data\_expr\_f(\_1,\_r1,\_pass,

904  boost::phoenix::ref(var\_map\_),

905  boost::phoenix::ref(error\_msgs\_))]

906  % ',')

907  > lit(']')

908  ;

909

910  range\_brackets\_int\_r.name("integer range expression pair, brackets");

911  range\_brackets\_int\_r

912  = lit('<') [\_val = empty\_range\_f(boost::phoenix::ref(error\_msgs\_))]

913  >> (

914  ( (lit("lower")

915  >> lit('=')

916  >> expression07\_g(\_r1)

917  [set\_int\_range\_lower\_f(\_val,\_1,\_pass,

918  boost::phoenix::ref(error\_msgs\_)) ])

919  >> -( lit(',')

920  >> lit("upper")

921  >> lit('=')

922  >> expression07\_g(\_r1)

923  [set\_int\_range\_upper\_f(\_val,\_1,\_pass,

924  boost::phoenix::ref(error\_msgs\_)) ] ) )

925  |

926  ( lit("upper")

927  >> lit('=')

928  >> expression07\_g(\_r1)

929  [set\_int\_range\_upper\_f(\_val,\_1,\_pass,

930  boost::phoenix::ref(error\_msgs\_)) ])

931  )

932  >> lit('>');

933

934  range\_brackets\_double\_r.name("real range expression pair, brackets");

935  range\_brackets\_double\_r

936  = lit('<') [\_val = empty\_range\_f(boost::phoenix::ref(error\_msgs\_))]

937  > (

938  ( (lit("lower")

939  > lit('=')

940  > expression07\_g(\_r1)

941  [set\_double\_range\_lower\_f(\_val,\_1,\_pass,

942  boost::phoenix::ref(error\_msgs\_)) ])

943  > -( lit(',')

944  > lit("upper")

945  > lit('=')

946  > expression07\_g(\_r1)

947  [set\_double\_range\_upper\_f(\_val,\_1,\_pass,

948  boost::phoenix::ref(error\_msgs\_)) ] ) )

949  |

950  ( lit("upper")

951  > lit('=')

952  > expression07\_g(\_r1)

953  [set\_double\_range\_upper\_f(\_val,\_1,\_pass,

954  boost::phoenix::ref(error\_msgs\_)) ])

955  )

956  > lit('>');

957

958  identifier\_r.name("identifier");

959  identifier\_r

960  %= identifier\_name\_r

961  [\_pass = validate\_identifier\_f(\_val,boost::phoenix::ref(error\_msgs\_))]

962  ;

963

964  identifier\_name\_r.name("identifier subrule");

965  identifier\_name\_r

966  %= lexeme[char\_("a-zA-Z")

967  >> \*char\_("a-zA-Z0-9\_.")]

968  ;

969

970  }

971  }

972

973

974 }

975 #endif

empty\_range::operator()

range operator()(std::stringstream &) const

**Definition:** var\_decls\_grammar\_def.hpp:522

validate\_decl\_constraints::operator()

bool operator()(const bool &allow\_constraints, const bool &declaration\_ok, const var\_decl &var\_decl, std::stringstream &error\_msgs) const

**Definition:** var\_decls\_grammar\_def.hpp:287

empty\_range

**Definition:** var\_decls\_grammar\_def.hpp:519

copy\_square\_cholesky\_dimension\_if\_necessary::operator()

void operator()(cholesky\_factor\_var\_decl &var\_decl) const

**Definition:** var\_decls\_grammar\_def.hpp:511

stan::gm::var\_decls\_grammar::identifier\_name\_r

boost::spirit::qi::rule< Iterator, std::string(), whitespace\_grammar< Iterator > > identifier\_name\_r

**Definition:** var\_decls\_grammar.hpp:78

stan::gm::var\_decls\_grammar::matrix\_decl\_r

boost::spirit::qi::rule< Iterator, matrix\_var\_decl(var\_origin), whitespace\_grammar< Iterator > > matrix\_decl\_r

**Definition:** var\_decls\_grammar.hpp:88

N\_

int N\_

**Definition:** mdivide\_left.hpp:20

set\_double\_range\_lower::result

**Definition:** var\_decls\_grammar\_def.hpp:625

stan::gm::data\_origin

const int data\_origin

**Definition:** ast.hpp:413

empty\_range\_f

boost::phoenix::function< empty\_range > empty\_range\_f

**Definition:** var\_decls\_grammar\_def.hpp:526

set\_int\_range\_lower\_f

boost::phoenix::function< set\_int\_range\_lower > set\_int\_range\_lower\_f

**Definition:** var\_decls\_grammar\_def.hpp:559

BOOST\_FUSION\_ADAPT\_STRUCT

BOOST\_FUSION\_ADAPT\_STRUCT(stan::gm::int\_var\_decl,(stan::gm::range, range\_)(std::string, name\_)(std::vector< stan::gm::expression >, dims\_)) BOOST\_FUSION\_ADAPT\_STRUCT(stan

**Definition:** var\_decls\_grammar\_def.hpp:22

stan::gm::parameter\_origin

const int parameter\_origin

**Definition:** ast.hpp:415

set\_int\_range\_lower::operator()

void operator()(range &range, const expression &expr, bool &pass, std::stringstream &error\_msgs) const

**Definition:** var\_decls\_grammar\_def.hpp:550

set\_double\_range\_upper

**Definition:** var\_decls\_grammar\_def.hpp:637

stan::gm::var\_decls\_grammar::row\_vector\_decl\_r

boost::spirit::qi::rule< Iterator, row\_vector\_var\_decl(var\_origin), whitespace\_grammar< Iterator > > row\_vector\_decl\_r

**Definition:** var\_decls\_grammar.hpp:118

copy\_square\_cholesky\_dimension\_if\_necessary::result

**Definition:** var\_decls\_grammar\_def.hpp:510

stan::gm::local\_origin

const int local\_origin

**Definition:** ast.hpp:418

validate\_int\_expr

**Definition:** var\_decls\_grammar\_def.hpp:528

set\_int\_range\_upper::operator()

void operator()(range &range, const expression &expr, bool &pass, std::stringstream &error\_msgs) const

**Definition:** var\_decls\_grammar\_def.hpp:564

validate\_decl\_constraints

**Definition:** var\_decls\_grammar\_def.hpp:283

stan::gm::range

**Definition:** ast.hpp:401

data\_only\_expression::operator()

bool operator()(const index\_op &x) const

**Definition:** var\_decls\_grammar\_def.hpp:220

validate\_identifier::identifier\_exists

bool identifier\_exists(const std::string &identifier) const

**Definition:** var\_decls\_grammar\_def.hpp:322

validate\_identifier::operator()

bool operator()(const std::string &identifier, std::stringstream &error\_msgs) const

**Definition:** var\_decls\_grammar\_def.hpp:474

stan::gm::var\_decls\_grammar::cholesky\_factor\_decl\_r

boost::spirit::qi::rule< Iterator, cholesky\_factor\_var\_decl(var\_origin), whitespace\_grammar< Iterator > > cholesky\_factor\_decl\_r

**Definition:** var\_decls\_grammar.hpp:43

validate\_identifier::result

**Definition:** var\_decls\_grammar\_def.hpp:310

stan::gm::var\_decls\_grammar::dims\_r

boost::spirit::qi::rule< Iterator, std::vector< expression >var\_origin), whitespace\_grammar< Iterator > > dims\_r

**Definition:** var\_decls\_grammar.hpp:63

stan::gm::print\_var\_origin

void print\_var\_origin(std::ostream &o, const var\_origin &vo)

**Definition:** ast\_def.cpp:944

stan::gm::var\_decls\_grammar::expression07\_g

expression07\_grammar< Iterator > expression07\_g

**Definition:** var\_decls\_grammar.hpp:36

data\_only\_expression::operator()

bool operator()(const variable &x) const

**Definition:** var\_decls\_grammar\_def.hpp:195

data\_only\_expression::operator()

bool operator()(const nil &) const

**Definition:** var\_decls\_grammar\_def.hpp:180

data\_only\_expression::error\_msgs\_

std::stringstream & error\_msgs\_

**Definition:** var\_decls\_grammar\_def.hpp:173

validate\_decl\_constraints::result

**Definition:** var\_decls\_grammar\_def.hpp:285

data\_only\_expression::operator()

bool operator()(const int\_literal &) const

**Definition:** var\_decls\_grammar\_def.hpp:183

copy\_square\_cholesky\_dimension\_if\_necessary\_f

boost::phoenix::function< copy\_square\_cholesky\_dimension\_if\_necessary > copy\_square\_cholesky\_dimension\_if\_necessary\_f

**Definition:** var\_decls\_grammar\_def.hpp:517

set\_int\_range\_lower::result

**Definition:** var\_decls\_grammar\_def.hpp:549

data\_only\_expression::operator()

bool operator()(const array\_literal &x) const

**Definition:** var\_decls\_grammar\_def.hpp:189

stan::gm::var\_decls\_grammar

**Definition:** var\_decls\_grammar.hpp:20

validate\_identifier::contains

static bool contains(const S &s, const T &x)

**Definition:** var\_decls\_grammar\_def.hpp:317

validate\_int\_data\_expr

**Definition:** var\_decls\_grammar\_def.hpp:577

validate\_double\_expr\_f

boost::phoenix::function< validate\_double\_expr > validate\_double\_expr\_f

**Definition:** var\_decls\_grammar\_def.hpp:620

stan::gm::function\_signatures

**Definition:** ast.hpp:106

stan::gm::var\_decls\_grammar::unit\_vector\_decl\_r

boost::spirit::qi::rule< Iterator, unit\_vector\_var\_decl(var\_origin), whitespace\_grammar< Iterator > > unit\_vector\_decl\_r

**Definition:** var\_decls\_grammar.hpp:123

validate\_double\_expr::operator()

bool operator()(const expression &expr, std::stringstream &error\_msgs) const

**Definition:** var\_decls\_grammar\_def.hpp:609

stan::math::integrate\_ode

std::vector< std::vector< typename stan::return\_type< T1, T2 >::type > > integrate\_ode(const F &f, const std::vector< T1 > y0, const double t0, const std::vector< double > &ts, const std::vector< T2 > &theta, const std::vector< double > &x, const std::vector< int > &x\_int, std::ostream \*msgs)

Return the solutions for the specified system of ordinary differential equations given the specified ...

**Definition:** integrate\_ode.hpp:61

set\_int\_range\_upper::result::type

void type

**Definition:** var\_decls\_grammar\_def.hpp:563

stan::gm::var\_decls\_grammar::vector\_decl\_r

boost::spirit::qi::rule< Iterator, vector\_var\_decl(var\_origin), whitespace\_grammar< Iterator > > vector\_decl\_r

**Definition:** var\_decls\_grammar.hpp:133

data\_only\_expression::operator()

bool operator()(const unary\_op &x) const

**Definition:** var\_decls\_grammar\_def.hpp:233

add\_var

**Definition:** var\_decls\_grammar\_def.hpp:239

set\_double\_range\_upper::result

**Definition:** var\_decls\_grammar\_def.hpp:639

copy\_square\_cholesky\_dimension\_if\_necessary

**Definition:** var\_decls\_grammar\_def.hpp:508

validate\_double\_expr::result

**Definition:** var\_decls\_grammar\_def.hpp:607

stan::gm::var\_decls\_grammar::corr\_matrix\_decl\_r

boost::spirit::qi::rule< Iterator, corr\_matrix\_var\_decl(var\_origin), whitespace\_grammar< Iterator > > corr\_matrix\_decl\_r

**Definition:** var\_decls\_grammar.hpp:53

validate\_int\_expr\_f

boost::phoenix::function< validate\_int\_expr > validate\_int\_expr\_f

**Definition:** var\_decls\_grammar\_def.hpp:545

data\_only\_expression::var\_map\_

variable\_map & var\_map\_

**Definition:** var\_decls\_grammar\_def.hpp:174

validate\_double\_expr

**Definition:** var\_decls\_grammar\_def.hpp:605

validate\_decl\_constraints\_f

boost::phoenix::function< validate\_decl\_constraints > validate\_decl\_constraints\_f

**Definition:** var\_decls\_grammar\_def.hpp:303

stan::gm::variable\_map

**Definition:** ast.hpp:439

validate\_identifier::result::type

bool type

**Definition:** var\_decls\_grammar\_def.hpp:310

stan::gm::var\_decls\_grammar::identifier\_r

boost::spirit::qi::rule< Iterator, std::string(), whitespace\_grammar< Iterator > > identifier\_r

**Definition:** var\_decls\_grammar.hpp:73

stan::gm::var\_decls\_grammar::var\_decls\_r

boost::spirit::qi::rule< Iterator, boost::spirit::qi::locals< bool >, std::vector< var\_decl >bool, var\_origin), whitespace\_grammar< Iterator > > var\_decls\_r

**Definition:** var\_decls\_grammar.hpp:145

validate\_int\_data\_expr::result::type

void type

**Definition:** var\_decls\_grammar\_def.hpp:579

empty\_range::result

**Definition:** var\_decls\_grammar\_def.hpp:521

stan::gm::var\_decls\_grammar::opt\_dims\_r

boost::spirit::qi::rule< Iterator, std::vector< expression >var\_origin), whitespace\_grammar< Iterator > > opt\_dims\_r

**Definition:** var\_decls\_grammar.hpp:93

stan::gm::var\_decls\_grammar::cholesky\_corr\_decl\_r

boost::spirit::qi::rule< Iterator, cholesky\_corr\_var\_decl(var\_origin), whitespace\_grammar< Iterator > > cholesky\_corr\_decl\_r

**Definition:** var\_decls\_grammar.hpp:48

stan::gm::var\_decls\_grammar::ordered\_decl\_r

boost::spirit::qi::rule< Iterator, ordered\_var\_decl(var\_origin), whitespace\_grammar< Iterator > > ordered\_decl\_r

**Definition:** var\_decls\_grammar.hpp:98

set\_int\_range\_upper

**Definition:** var\_decls\_grammar\_def.hpp:561

validate\_identifier

**Definition:** var\_decls\_grammar\_def.hpp:305

data\_only\_expression::operator()

bool operator()(const integrate\_ode &x) const

**Definition:** var\_decls\_grammar\_def.hpp:210

stan::gm::var\_decls\_grammar::double\_decl\_r

boost::spirit::qi::rule< Iterator, double\_var\_decl(var\_origin), whitespace\_grammar< Iterator > > double\_decl\_r

**Definition:** var\_decls\_grammar.hpp:68

validate\_identifier::const\_fun\_name\_set\_

std::set< std::string > const\_fun\_name\_set\_

**Definition:** var\_decls\_grammar\_def.hpp:307

stan::gm::var\_decls\_grammar::var\_decl\_r

boost::spirit::qi::rule< Iterator, boost::spirit::qi::locals< bool >, var\_decl(bool, var\_origin), whitespace\_grammar< Iterator > > var\_decl\_r

**Definition:** var\_decls\_grammar.hpp:139

data\_only\_expression::operator()

bool operator()(const fun &x) const

**Definition:** var\_decls\_grammar\_def.hpp:214

validate\_int\_expr::operator()

void operator()(const expression &expr, bool &pass, std::stringstream &error\_msgs) const

**Definition:** var\_decls\_grammar\_def.hpp:532

set\_double\_range\_lower::result::type

void type

**Definition:** var\_decls\_grammar\_def.hpp:625

validate\_identifier\_f

boost::phoenix::function< validate\_identifier > validate\_identifier\_f

**Definition:** var\_decls\_grammar\_def.hpp:505

set\_double\_range\_lower::operator()

void operator()(range &range, const expression &expr, bool &pass, std::stringstream &error\_msgs) const

**Definition:** var\_decls\_grammar\_def.hpp:626

set\_int\_range\_lower::result::type

void type

**Definition:** var\_decls\_grammar\_def.hpp:549

var\_decls\_grammar.hpp

stan::gm::var\_decls\_grammar::range\_brackets\_double\_r

boost::spirit::qi::rule< Iterator, range(var\_origin), whitespace\_grammar< Iterator > > range\_brackets\_double\_r

**Definition:** var\_decls\_grammar.hpp:108

data\_only\_expression::data\_only\_expression

data\_only\_expression(std::stringstream &error\_msgs, variable\_map &var\_map)

**Definition:** var\_decls\_grammar\_def.hpp:175

set\_double\_range\_upper::result::type

void type

**Definition:** var\_decls\_grammar\_def.hpp:639

set\_int\_range\_upper\_f

boost::phoenix::function< set\_int\_range\_upper > set\_int\_range\_upper\_f

**Definition:** var\_decls\_grammar\_def.hpp:573

stan::gm::int\_var\_decl

**Definition:** ast.hpp:455

set\_double\_range\_lower\_f

boost::phoenix::function< set\_double\_range\_lower > set\_double\_range\_lower\_f

**Definition:** var\_decls\_grammar\_def.hpp:635

data\_only\_expression::operator()

bool operator()(const binary\_op &x) const

**Definition:** var\_decls\_grammar\_def.hpp:229

set\_double\_range\_upper::operator()

void operator()(range &range, const expression &expr, bool &pass, std::stringstream &error\_msgs) const

**Definition:** var\_decls\_grammar\_def.hpp:640

validate\_int\_data\_expr::result

**Definition:** var\_decls\_grammar\_def.hpp:579

ast.hpp

set\_int\_range\_upper::result

**Definition:** var\_decls\_grammar\_def.hpp:563

stan::gm::var\_decls\_grammar::range\_brackets\_int\_r

boost::spirit::qi::rule< Iterator, range(var\_origin), whitespace\_grammar< Iterator > > range\_brackets\_int\_r

**Definition:** var\_decls\_grammar.hpp:113

stan::gm::transformed\_data\_origin

const int transformed\_data\_origin

**Definition:** ast.hpp:414

stan::gm::is\_nil

bool is\_nil(const expression &e)

**Definition:** ast\_def.cpp:768

stan::gm::var\_decls\_grammar::positive\_ordered\_decl\_r

boost::spirit::qi::rule< Iterator, positive\_ordered\_var\_decl(var\_origin), whitespace\_grammar< Iterator > > positive\_ordered\_decl\_r

**Definition:** var\_decls\_grammar.hpp:103

set\_double\_range\_upper\_f

boost::phoenix::function< set\_double\_range\_upper > set\_double\_range\_upper\_f

**Definition:** var\_decls\_grammar\_def.hpp:649

validate\_identifier::reserved\_word\_set\_

std::set< std::string > reserved\_word\_set\_

**Definition:** var\_decls\_grammar\_def.hpp:306

validate\_double\_expr::result::type

bool type

**Definition:** var\_decls\_grammar\_def.hpp:607

stan::gm::var\_decls\_grammar::error\_msgs\_

std::stringstream & error\_msgs\_

**Definition:** var\_decls\_grammar.hpp:31

stan::gm::var\_decls\_grammar::cov\_matrix\_decl\_r

boost::spirit::qi::rule< Iterator, cov\_matrix\_var\_decl(var\_origin), whitespace\_grammar< Iterator > > cov\_matrix\_decl\_r

**Definition:** var\_decls\_grammar.hpp:58

validate\_int\_expr::result

**Definition:** var\_decls\_grammar\_def.hpp:530

common\_adaptors\_def.hpp

stan::gm::var\_decls\_grammar::int\_decl\_r

boost::spirit::qi::rule< Iterator, int\_var\_decl(var\_origin), whitespace\_grammar< Iterator > > int\_decl\_r

**Definition:** var\_decls\_grammar.hpp:83

stan::gm::INT\_T

const int INT\_T

**Definition:** ast.hpp:65

stan::gm::var\_decls\_grammar::expression\_g

expression\_grammar< Iterator > expression\_g

**Definition:** var\_decls\_grammar.hpp:34

stan::gm::transformed\_parameter\_origin

const int transformed\_parameter\_origin

**Definition:** ast.hpp:416

empty\_range::result::type

range type

**Definition:** var\_decls\_grammar\_def.hpp:521

data\_only\_expression::operator()

bool operator()(const double\_literal &) const

**Definition:** var\_decls\_grammar\_def.hpp:186

validate\_int\_data\_expr::operator()

void operator()(const expression &expr, int var\_origin, bool &pass, variable\_map &var\_map, std::stringstream &error\_msgs) const

**Definition:** var\_decls\_grammar\_def.hpp:581

validate\_int\_data\_expr\_f

boost::phoenix::function< validate\_int\_data\_expr > validate\_int\_data\_expr\_f

**Definition:** var\_decls\_grammar\_def.hpp:603

validate\_identifier::validate\_identifier

validate\_identifier()

**Definition:** var\_decls\_grammar\_def.hpp:328

validate\_int\_expr::result::type

void type

**Definition:** var\_decls\_grammar\_def.hpp:530

validate\_identifier::reserve

void reserve(const std::string &w)

**Definition:** var\_decls\_grammar\_def.hpp:312

set\_double\_range\_lower

**Definition:** var\_decls\_grammar\_def.hpp:623

validate\_decl\_constraints::result::type

bool type

**Definition:** var\_decls\_grammar\_def.hpp:285

add\_var\_f

boost::phoenix::function< add\_var > add\_var\_f

**Definition:** var\_decls\_grammar\_def.hpp:280

stan::gm::var\_decls\_grammar::simplex\_decl\_r

boost::spirit::qi::rule< Iterator, simplex\_var\_decl(var\_origin), whitespace\_grammar< Iterator > > simplex\_decl\_r

**Definition:** var\_decls\_grammar.hpp:128

stan::gm::var\_decls\_grammar::var\_map\_

variable\_map & var\_map\_

**Definition:** var\_decls\_grammar.hpp:30

stan::gm::var\_origin

int var\_origin

**Definition:** ast.hpp:411

copy\_square\_cholesky\_dimension\_if\_necessary::result::type

void type

**Definition:** var\_decls\_grammar\_def.hpp:510

add\_var::operator()

void operator()(R &var\_decl\_result, const T &var\_decl, variable\_map &vm, bool &pass, const var\_origin &vo, std::ostream &error\_msgs) const

**Definition:** var\_decls\_grammar\_def.hpp:244

data\_only\_expression

**Definition:** var\_decls\_grammar\_def.hpp:172

add\_var::result::type

void type

**Definition:** var\_decls\_grammar\_def.hpp:241

add\_var::result

**Definition:** var\_decls\_grammar\_def.hpp:241

M\_

int M\_

**Definition:** mdivide\_left.hpp:19

set\_int\_range\_lower

**Definition:** var\_decls\_grammar\_def.hpp:547


---

[ Stan Home Page ]

*© 2011–2014,
Stan Development Team.*
